# The Warburg hypothesis and the emergence of the mitochondrial metabolic theory of cancer

**DOI:** 10.1007/s10863-025-10059-w

**Published:** 2025-04-08

**Authors:** Thomas N. Seyfried, Derek C. Lee, Tomas Duraj, Nathan L. Ta, Purna Mukherjee, Michael Kiebish, Gabriel Arismendi-Morillo, Christos Chinopoulos

**Affiliations:** 1 Biology Department, Boston College, 140 Commonwealth Ave, Chestnut Hill, Boston, MA 02467 USA; 2 BPGbio, Framingham, MA 01701 USA; 3 Facultad de Medicina, Instituto de Investigaciones Biológicas, Universidad del Zulia, Maracaibo, Venezuela; 4 Department of Medicine, Faculty of Health Sciences, University of Deusto, Bilbao (Bizkaia), Spain; 5 Department of Medical Biochemistry, Semmelweis University, Budapest, 1094 Hungary

**Keywords:** Oxidative phosphorylation, Substrate level phosphorylation, Oxygen consumption, Lactate, Succinate, Somatic mutations, Lipid droplets, Cardiolipin

## Abstract

Otto Warburg originally proposed that cancer arose from a two-step process. The first step involved a chronic insufficiency of mitochondrial oxidative phosphorylation (OxPhos), while the second step involved a protracted compensatory energy synthesis through lactic acid fermentation. His extensive findings showed that oxygen consumption was lower while lactate production was higher in cancerous tissues than in non-cancerous tissues. Warburg considered both oxygen consumption and extracellular lactate as accurate markers for ATP production through OxPhos and glycolysis, respectively. Warburg’s hypothesis was challenged from findings showing that oxygen consumption remained high in some cancer cells despite the elevated production of lactate suggesting that OxPhos was largely unimpaired. New information indicates that neither oxygen consumption nor lactate production are accurate surrogates for quantification of ATP production in cancer cells. Warburg also did not know that a significant amount of ATP could come from glutamine-driven mitochondrial substrate level phosphorylation in the glutaminolysis pathway with succinate produced as end product, thus confounding the linkage of oxygen consumption to the origin of ATP production within mitochondria. Moreover, new information shows that cytoplasmic lipid droplets and elevated aerobic lactic acid fermentation are both biomarkers for OxPhos insufficiency. Warburg’s original hypothesis can now be linked to a more complete understanding of how OxPhos insufficiency underlies dysregulated cancer cell growth. These findings can also address several questionable assumptions regarding the origin of cancer thus allowing the field to advance with more effective therapeutic strategies for a less toxic metabolic management and prevention of cancer.

## Introduction

Otto Warburg originally proposed that all cancers, regardless of animal species or tissue origin, arose from chronic disturbances of cellular respiration that would diminish ATP production through oxidative phosphorylation (OxPhos) (Warburg 1931, 1956a, 1956b, 1969). Dysregulated cell growth (cancer) would occur, however, only if ATP production through fermentation could compensate for the insufficient ATP production through OxPhos. These processes must occur chronically as acute OxPhos inhibition would kill the cell rather than transform it to a cancer cell. Based on earlier studies in sea urchins and in slice preparations from normal tissues, Warburg considered oxygen consumption as an accurate marker for ATP production through respiration and viewed lactate production as the sole marker for ATP production through fermentation (Warburg 1931; Krebs 1981). Warburg also used the “Meyerhof Quotient” as a quantitative estimate for assessing the effectiveness of respiration in preventing aerobic fermentation (Krebs 1981; Warburg 1931). Using units of oxygen consumption as the denominator and anaerobic glycolysis minus aerobic glycolysis in lactate units as the numerator, Warburg proposed that respiration in cancer cells was insufficient in reducing aerobic fermentation (glycolysis). In other words, the high aerobic glycolytic rate seen in all major cancers resulted as an effect of OxPhos insufficiency. Warburg held his view that cancer arose from chronic OxPhos insufficiency even when evaluating cancer cells where oxygen consumption was similar to that in non-cancerous cells (Warburg 1931).

Sidney Weinhouse, another preeminent researcher in cancer metabolism, seriously challenged Warburg’s hypothesis. Tumor cells that maintained high oxygen consumption was evidence to Weinhouse that OxPhos insufficiency could not explain the origin of cancer according to Warburg’s hypothesis (Weinhouse 1956, 1976). He also claimed that oxygen consumption in various tumors was, by and large, similar to that in non-neoplastic cells and tissues as long as differences in basal metabolic rate among different species (rat, mouse, and humans) were ignored (Weinhouse 1956). However, without recognizing the inter-species differences in basal metabolic rate, which is about seven-fold higher in mice than in humans (Terpstra 2001; Porter and Brand 1993), comparisons of tumor oxygen consumption that disregard differences in host metabolic rates are difficult to interpret if not meaningless (Burk and Schade 1956).

As an alternative to Warburg’s hypothesis, Weinhouse suggested that normal respiration and a normal Pasteur effect were incapable of eliminating the high glycolytic rate seen in some cancer cells making abnormally high glycolysis, rather than OxPhos insufficiency, as the key issue in the origin of cancers (Weinhouse 1956). Despite Warburg’s rebuttal to Weinhouse’s misunderstanding of concepts and Burk and Schade’s credible counter evidence against Weinhouse’s misinterpretation of data, Weinhouse continued to believe that mitochondria and ATP production through OxPhos were largely uncompromised in cancer cells (Warburg 1956b; Weinhouse 1976; Burk and Schade 1956).

Although Warburg’s central hypothesis was supported from the information presented in Alan Aisenberg’s monograph on the glycolysis and respiration in tumors and from Sidney Colowick’s review of the evidence (Aisenberg 1961; Colowick 1961), many investigators of cancer metabolism eventually sided with Weinhouse’s arguments in considering OxPhos as largely unimpaired in cancer and that gene-linked abnormalities in the regulation of glycolysis were mostly responsible for the growth of cancer cells (Zu and Guppy 2004; Koppenol et al. 2011; Moreno-Sanchez et al. 2014; DeBerardinis and Chandel 2016; Vander Heiden et al. 2009; Liberti and Locasale 2016). Whether the mitochondria function normally or abnormally in cancer cells has not yet been resolved despite research spanning almost a century (Aisenberg 1961; Pedersen 1978; Seyfried et al. 2020; Warburg 1925; Colowick 1961). Several questionable assumptions regarding the origin of energy production and the role of gene mutations in cancer are listed below that have confounded data interpretation and have delayed acceptance of the mitochondrial metabolic theory based on Warburg’s original hypothesis**.**

### Questionable Assumption 1. Oxygen consumption is an accurate marker for ATP production through OxPhos in cancer cells

 Based first on the work of Meyerhof and later on the work of Krebs and co-workers in rat liver homogenates, Warburg assumed that seven moles of ATP could be formed when one mole of oxygen was consumed in the respiration of either normal cells or cancer cells (H. A. Krebs et al. 1953; Warburg 1931). Weinhouse made similar assumptions emphasizing that not all cancer cells had reduced oxygen consumption and could not therefore have insufficient OxPhos compared to normal cells and tissues as Warburg claimed (Weinhouse 1956; 1976). Koppenol et al., later claimed that Warburg’s error in thinking that OxPhos was lower in cancer tissue than in normal tissue was due to hypoxia in the thickness of Warburg’s tumor tissue slice preparations (≥ 400 mm) (Koppenol et al. 2011). However, earlier findings by Nelicia Mayer showed that oxygen consumption was lower in both tissue slices and in homogenates of tumor tissue than of normal tissues and homogenates, thus ruling out tissue slice thickness as a compelling argument against Warburg’s conclusions (Mayer 1944). Moreover, claims of unimpaired OxPhos function in tumor cells based solely on short-term respirometry (less than two hours) experiments must be viewed with caution according to the recent findings of Duraj and others (Duraj et al. 2021; D. C. Lee et al. 2024; Schmidt et al. 2021). Indeed, changes in oxygen consumption under specific mitochondrial targeting (such as oligomycin-linked oxygen consumption rate) are subject to many caveats and are not functionally informative on the sufficiency or insufficiency of OxPhos for viability and/or proliferation (Duraj et al. 2021; D. C. Lee et al. 2024).

It is important for us to emphasize that the reliance on oxygen consumption rate (OCR) as a marker for OxPhos efficiency in tumor cells is just as ambiguous today as it was for Warburg 100 years ago. We recently used a novel experimental design in measuring OCR in tandem with ATP-dependent bioluminescence to show that oxygen consumption is not a reliable measure for ATP production through OxPhos in mouse and human glioma cells (D. C. Lee et al. 2024). While many recent studies have assumed that OCR is an accurate measure of efficient OxPhos, none of these studies measured OCR in tandem with ATP-dependent bioluminescence or considered mitochondrial substrate level phosphorylation as a second, OxPhos independent source, of ATP production in tumor cell mitochondria (Moreno-Sanchez et al. 2009; Vaupel and Mayer 2012; Margetis 2023; Ward and Thompson 2012; Koppenol et al. 2011; Liberti and Locasale 2016; Janiszewska et al. 2012; Shiratori et al. 2019; Rodrigues et al. 2016; Saha et al. 2022; P. Herst et al. 2024; Vaupel and Multhoff 2021). Hence, caution is needed in assuming that OCR is an accurate measure of ATP production through OxPhos in tumor cells using short-term respirometry and without also considering glutamine-driven mitochondrial substrate level phosphorylation as a compounding variable for ATP production (Lee et al. 2024; Duraj et al. 2024, 2021).

Data from several studies also showed that oxygen and the electron transport chain (ETC) can be used for metabolic activities other than ATP production through OxPhos in tumor cells (Joshi and Patel 2023; Leznev et al. 2013; Hall et al. 2013; P. M. Herst and Berridge 2007; Arcos et al. 1969; Ramanathan et al. 2005; Seyfried et al. 2020). Oxygen-derived reactive oxygen species (ROS), which reduce OxPhos function and contribute to nuclear genomic mutations, are generally greater in cancer cells than in normal cells (Bartesaghi et al. 2015; Zorov et al. 2014; Hervouet and Godinot 2006; Rodic and Vincent 2018; Lemarie and Grimm 2011). Excess ROS, in conjunction with proton leakage, can also uncouple the electrochemical gradient thus reducing ATP production through the ATP synthase (Valle et al. 2010; Villalobo and Lehninger 1979). New studies now suggest that ATP production through OxPhos is neither necessary nor sufficient for brain tumor cell growth in the absence of fermentable fuels (D. C. Lee et al. 2024). Indeed, no evidence has been presented showing any tumor cell that can grow in the absence of fermentable fuels regardless of oxygen consumption. When viewed collectively, the available evidence challenges the assumption that oxygen consumption is an accurate marker for ATP production through OxPhos in cancer cells.

### Questionable Assumption 2. Lactic acid is an accurate marker for ATP production through glycolysis in cancer cells

Based on findings from his laboratory, Warburg assumed that one mole of ATP could be formed when one mole of lactic acid was produced from glucose fermentation in cancer cells (Warburg 1931; 1956a). While this would be correct for most normal cells and tissues, it would not be correct for cancer cells and tissues. In contrast to the pyruvate kinase isoform (PKM1), which produces ATP through substrate level phosphorylation in the glycolytic pathway, the low affinity pyruvate kinase 2 isoform (PKM2) is also active in cancer cells where significant lactate can be produced independent of ATP production (Vander Heiden et al. 2010; Israelsen et al. 2013; Chinopoulos 2020; D. C. Lee et al. 2024). As tumor cells predominantly contain mixtures of both the PKM1 & the PKM2 isoforms, it becomes difficult to accurately estimate ATP production through either aerobic or anaerobic glucose-linked fermentation based solely on lactate production (D. C. Lee et al. 2024). In addition to alleviating reductive stress, the activity of PKM2 generates an upstream metabolic traffic jam that will divert the intermediate metabolites of glycolysis to anabolic processes (Chinopoulos 2020). Lactic acid could be an accurate marker for ATP production through glycolysis if 1) the ratio of PKM1:PKM2 is determined, and 2) the ratio of tetramerized PKM2 to dimerized PKM2 is determined. No studies have yet made these determinations to our knowledge. Some lactate might also be produced from glutamine, but the amount is negligible compared to the amount produced from glucose (Ta and Seyfried 2015; Seyfried et al. 2020; DeBerardinis et al. 2007). The inaccuracy of extracellular lactate production as a marker for ATP production through glycolysis viewed together with the inaccuracy of oxygen consumption rate as a predictor of ATP production through OxPhos make the conclusions from either Warburg or Weinhouse on ATP production in cancer cells based on the “Meyerhof Quotient” as largely inaccurate (H. Krebs 1981; Koppenol et al. 2011; Warburg 1931).

### Questionable Assumption 3. OxPhos and lactic acid fermentation are the only sources of ATP production in cancer cells

In addition to OxPhos and glycolysis, the original studies of Kaufman et al., Hift et al., and Sanadi et al., showed that ATP could also be produced through a substrate level phosphorylation reaction catalyzed by succinyl CoA synthetase in the TCA cycle (Sanadi et al. 1956; Kaufman et al. 1953; Hift et al. 1953). Assumption three is questionable because it fails to recognize the significant contribution of glutamine-driven mitochondrial substrate level phosphorylation (mSLP) through the glutaminolysis pathway as an additional source of ATP production in cancer cells (Auger et al. 2021; D. C. Lee et al. 2024; Chinopoulos and Seyfried 2018; Flores et al. 2018; Doczi et al. 2023; Gao et al. 2016; Ravasz et al. 2024). Just as lactate is the predominant end-product of glucose fermentation produced through cytosolic substrate level phosphorylation in the Embden-Meyerhof-Parnas pathway, succinate is the predominant end-product of glutamine fermentation produced through mitochondrial substrate level phosphorylation in the glutaminolysis pathway (Chinopoulos 2019; Ravasz et al. 2024; D. C. Lee et al. 2024; Slaughter et al. 2016). It is well-documented that glucose and glutamine fermentation can compensate for transient OxPhos inefficiency during prolonged diving (Hochachka et al. 1975), ischemia (Taegtmeyer 1978; J. Zhang et al. 2018; Chinopoulos 2019), hemorrhagic shock (Taghavi et al. 2022; Slaughter et al. 2016), and high-intensity muscle exercise (Reddy et al. 2020). The accumulation of lactate and succinate are the biomarkers for compensatory ATP maintenance through SLP in the cytosol and in the mitochondria, respectively (Chinopoulos 2019; Reddy et al. 2020; D. C. Lee et al. 2024). It is noteworthy, however, that the extracellular accumulation of lactate and succinate ceases following the resumption of OxPhos activity in nonneoplastic cells indicating that their linkage to ATP maintenance through fermentation is transient (Reddy et al. 2020; Hochachka et al. 1975).

In contrast to the transient extracellular accumulation of fermentation end products seen in oxygen deprived normal cells and tissues, many tumor cells chronically produce lactate and succinate even in the presence of oxygen (D. C. Lee et al. 2024; C. C. Kuo et al. 2022; Selak et al. 2005; Zhao et al. 2017). Accumulation of extracellular lactate and succinate will contribute to the acidification of the cancer microenvironment, thus contributing to tumor progression (Seyfried et al. 2022; Bayley and Devilee 2010; C. C. Kuo et al. 2022). In other words, the continuous elevation of mitochondrial SLP with succinate production and cytosolic SLP with lactate production in the presence of oxygen are both effects of chronic OxPhos insufficiency, i.e., the bioenergetic signature of most, if not all, major cancers.

Neither Warburg nor Weinhouse knew that significant ATP could be produced through glutamine-driven mSLP in the glutaminolysis pathway with succinate generated as end product, thus confounding the linkage of oxygen consumption to the origin of ATP production within tumor cell mitochondria. While glutamine has been largely recognized as an anaplerotic respiratory fuel for growth (DeBerardinis et al. 2007), new findings show that glutamine can also be fermented for ATP production via mitochondrial SLP (Doczi et al. 2023; Ravasz et al. 2024; D. C. Lee et al. 2024). By sustaining the activity of the oligomycin-sensitive F1-F0-ATPase operating in reverse, mitochondrial SLP activity during hypoxia in normal cells or normoxia/hypoxia in cancer cells, will prevent the reverse operation of the adenine nucleotide transporter (ANT) thus preventing the life-threatening consumption of cytosolic ATP reserves (Zhdanov et al. 2017; Chinopoulos and Adam-Vizi 2010; Chinopoulos et al. 2010). While both the electron transport chain and oxygen consumption can influence the efficiency of mitochondrial SLP, this influence appears independent of ATP production through OxPhos (Chinopoulos 2011).

We recently suggested that ATP could be produced in glioblastoma through mitochondrial substrate-level phosphorylation (mSLP) in the glutaminolysis pathway (Seyfried et al. 2020; Chinopoulos and Seyfried 2018; D. C. Lee et al. 2024). Previous studies showed that oxygen-independent mSLP was a major source of ATP content necessary for the growth of the parasite *Trypanosoma brucei* (Bochud-Allemann and Schneider 2002; Taleva et al. 2023). Moreover, mSLP could rescue proliferation in respiration-impaired yeast by maintaining the mitochondrial membrane potential (Schwimmer et al. 2005). Previous studies in non-neural cancer cells also provided evidence for a role of mitochondrial SLP in driving tumor growth (Gao et al. 2016). Further studies will be needed to document the role of mitochondrial SLP as a source of ATP production in cancer.

**Fig. 1:**
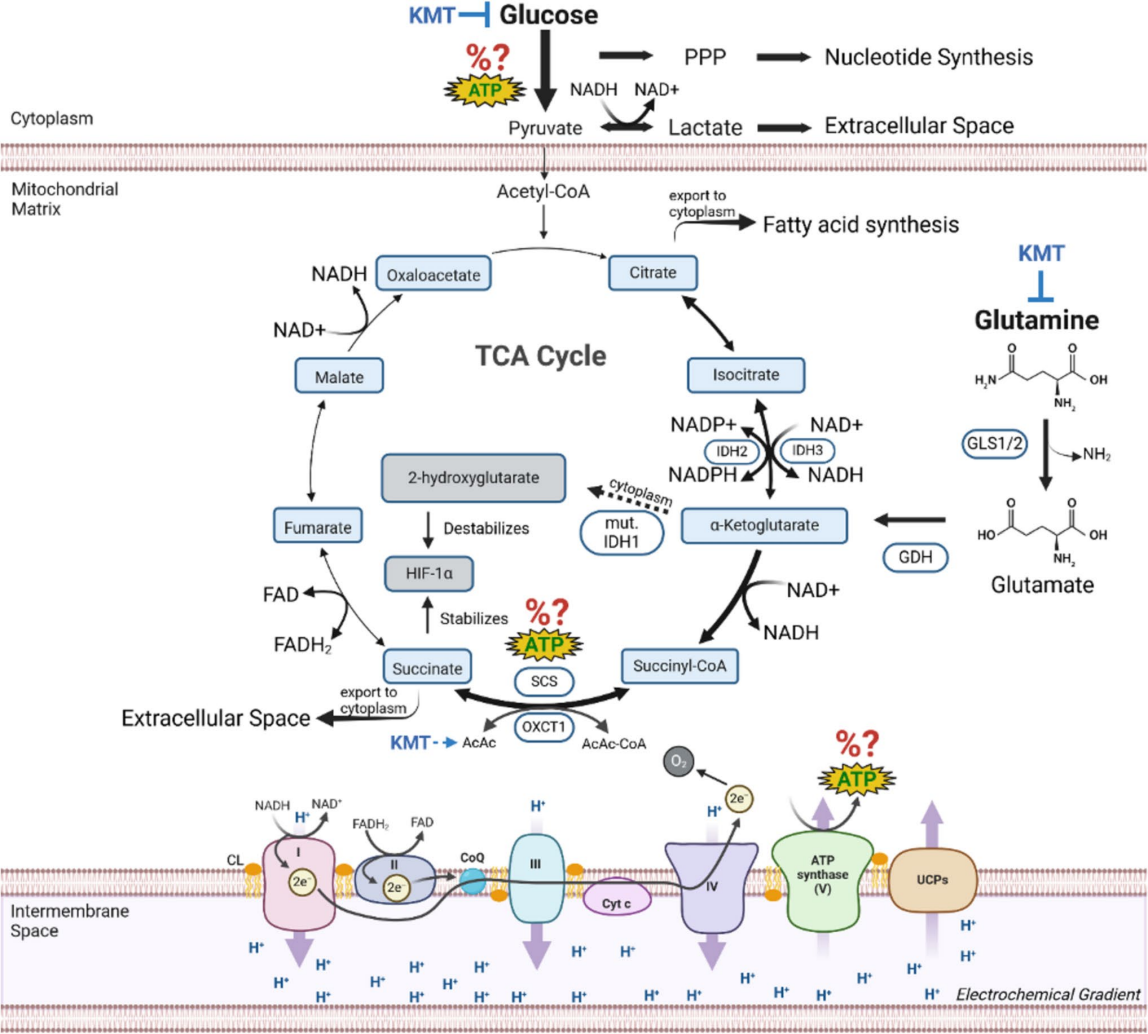
ATP production and vulnerability of cancer cells to metabolic stress. Besides OxPhos and cytosolic substrate level phosphorylation (cSLP) in the pay-off phase of glycolysis, ATP can also be produced through glutamine-driven mitochondrial substrate level phosphorylation (mSLP) in the glutaminolysis pathway (Chinopoulos and Seyfried 2018; Seyfried et al. 2020; D. C. Lee et al. 2024; Doczi et al. 2023). The glutaminolysis pathway becomes elevated in tumor cells with inefficient OxPhos that also express the dimeric PKM2 isoform, which produces less ATP through glycolysis than does the PKM1 isoform. The percentage of ATP produced through OxPhos, mSLP, and cytosolic SLP would be context dependent in any given tumor and cannot therefore be accurately measured; hence the red question marks (?). Besides increasing the energetic efficiency of normal cells, by increasing the ΔG’ of ATP hydrolysis, the elevation of circulating ketone bodies (β-hydroxybutyrate and acetoacetate) through ketogenic metabolic therapy (KMT) could indirectly reduce ATP synthesis through the succinyl-CoA synthetase (SCS) reaction by diverting CoA to acetoacetate. The *IDH1* mutation, present in some gliomas, could further reduce ATP synthesis through mSLP by increasing synthesis of 2-hydroxyglutarate from α-ketoglutarate and thus reducing the succinyl-CoA substrate for the SCS reaction (Seyfried et al. 2021). In addition to its potential effect in reducing glutaminolysis, 2-hydroxyglutarate can also target multiple HIF1α-responsive genes and enzymes in the glycolysis pathway thus limiting synthesis of metabolites and one-carbon metabolism needed for rapid tumour growth (K. Zhang et al. 2017; Chinopoulos and Seyfried 2018; Seyfried et al. 2020; Chesnelong et al. 2014). The down regulation of HIF1α-regulated lactate dehydrogenase A (LDHA), through the action of both KMT and the IDH1 mutation, would reduce extracellular lactate and succinate levels thus reducing microenvironment inflammation and tumour cell invasion. Hence, the simultaneous inhibition of cytosolic and mitochondrial SLP, while the body is in a state of therapeutic ketosis (GKI 2.0 or below), will stress the majority of signaling pathways necessary for rapid tumor growth. Arrow thickness denotes higher relative flux of the glycolysis and glutaminolysis pathways as previously predicted (E. A. Newsholme and Board 1991). PPP = pentose phosphate pathway; KMT = ketogenic metabolic therapy; GLS = glutaminase; GDH = glutamate dehydrogenase; IDH = isocitrate dehydrogenase; SCS = succinyl-CoA synthetase; OXCT1 = succinyl-CoA:3- ketoacid CoA transferase; HIF1α = hypoxia-inducible factor 1 subunit alpha; UCPs = uncoupling proteins; CoQ = coenzyme Q; Cyt c = cytochrome c; AcAc = acetoacetate; AcAc-CoA = acetoacetyl-CoA; NAD + = nicotinamide adenine dinucleotide; NADH = nicotinamide adenine dinucleotide, reduced; NADP + = nicotinamide adenine dinucleotide phosphate; NADPH = nicotinamide adenine dinucleotide phosphate, reduced; CL = cardiolipin. Figure created using BioRender

It is important to recognize that glutamine consumption in many cancer cells can support both metabolite synthesis through the reductive carboxylation pathway as well as ATP production through mSLP (oxidative decarboxylation pathway) thus maintaining growth and viability (J. Jin et al. 2023; Scott et al. 2011; Jiang et al. 2019; Wise et al. 2008). The failure to recognize mitochondrial SLP as a major source of ATP production continues to confuse the issue of mitochondrial function in cancer and how tumor cells can survive and grow with minimal contributions from either OxPhos or glycolysis. Figure 1 illustrates the three sources of ATP production in cancer cells. Although cytosolic and mitochondrial SLP are necessary and sufficient for driving cancer cell growth, new findings in mouse and human glioma cells show that ATP production through OxPhos is neither necessary nor sufficient (D. C. Lee et al. 2024). A better understanding of metabolic control logic will be needed to more accurately quantify the amount of ATP produced from each of the three known sources needed for tumor cell growth due to the unpredictable metabolic flux of glucose and glutamine fuel utilization supporting each source of ATP production (E. A. Newsholme and Board 1991). Hence, question marks (?) are placed next to each ATP source in Fig. 1**.**

It is important to mention that we avoid using the term “Warburg effect” in our review as Warburg considered aerobic lactic acid fermentation as too labile and too dependent on external conditions (Warburg 1956a). Cytosolic SLP is not the same as the Warburg effect since SLP is independent of the end-product or presence of oxygen. The Warburg effect is typically defined both by the end-product (lactate) and the presence of oxygen (aerobic lactic acid fermentation) because persistently elevated lactate production would be unexpected in normoxia. Racker coined the term and simply considered the Warburg effect as an expression of high aerobic glycolysis in tumors (Racker 1972). The term has generated significant confusion in the cancer field as aerobic glycolysis occurs in most oxygenated normal cells where pyruvate is produced as the end product of the Embden-Meyerhof-Parnas pathway. We prefer the term “increased cytosolic substrate level phosphorylation”, rather than Warburg effect, to describe the mechanism of ATP production in the cell cytosol. Moreover, recent evidence suggests that lactic acid fermentation, i.e., the lactic acid dehydrogenase catalyzed reaction, is not required for proliferation (Zdralevic et al. 2018; Hefzi et al. 2025). While the impact on tumorigenicity has not been fully established, and this phenomenon has not been documented without genetic editing, it is important to note that cytosolic SLP provides ATP, not the lactate production contributing to NAD + regeneration. The “Warburg effect” may be dispensable for tumor cell proliferation under specific conditions, provided that the NAD + /NADH ratio is maintained (Hefzi et al. 2025). Several metabolic pathways have been identified to operate in favour of NAD^+^ regeneration (i.e., oxidizing NADH) or sustenance (i.e., inhibition of NAD^+^ reduction to NADH) when OxPhos is insufficient in cancer cells (Chinopoulos 2024; Birsoy et al. 2015; Luengo et al. 2021). These findings attest to the critical importance of maintaining a sufficiently high NAD^+^/NADH ratio supporting cancer cell viability under OxPhos insufficiency. Most importantly, both increased cytosolic and mitochondrial SLP (ATP-generating) appear to be necessary prerequisites for dysregulated tumor cell growth when OxPhos efficiency is compromised.

### Questionable Assumption 4. The number, structure, and function of mitochondria are similar in tumor tissue and in non-cancerous tissues

**Fig. 2:**
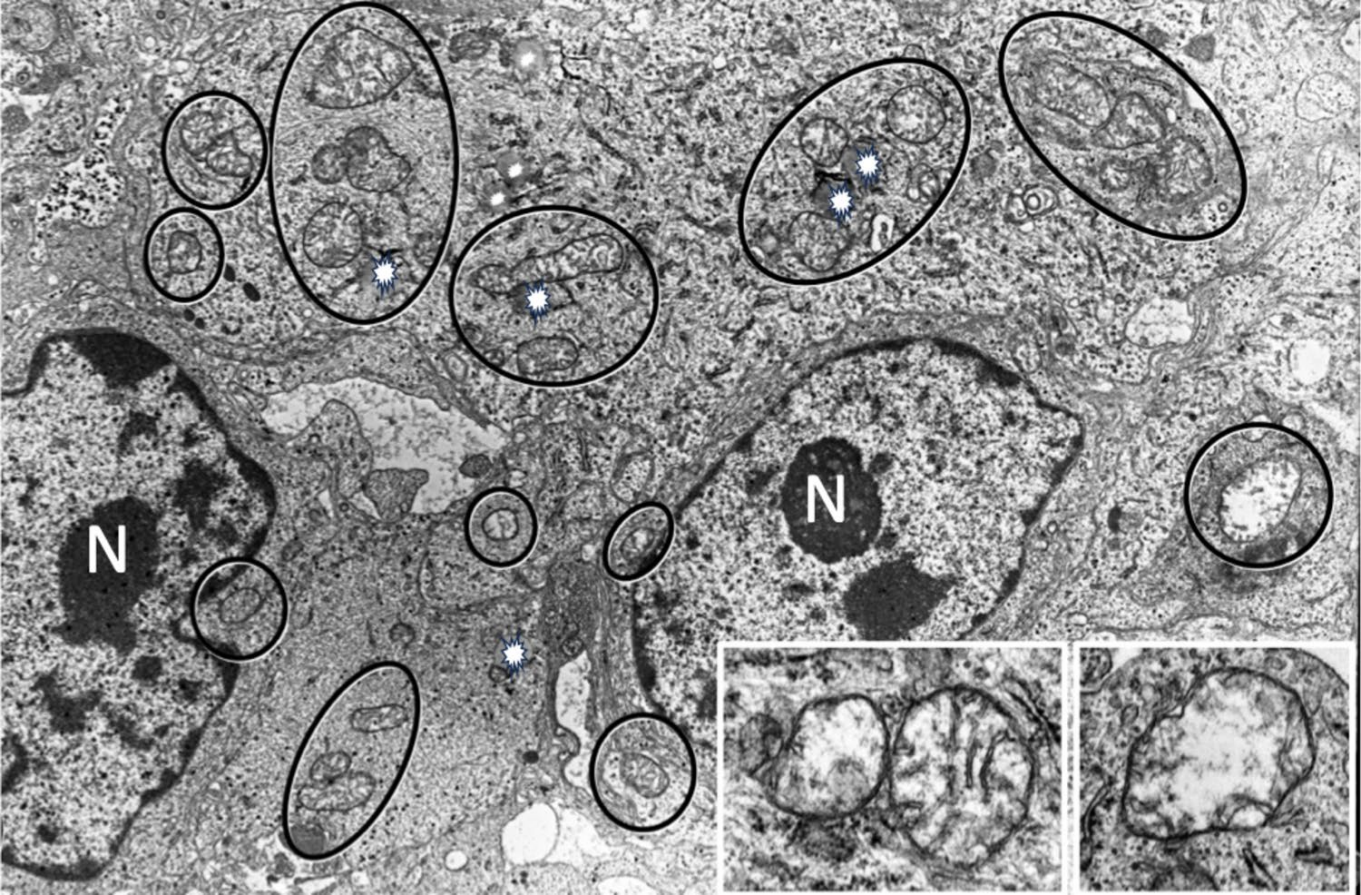
Abnormal mitochondria and lipid droplets in glioblastoma. Transmission electron microscopy (TEM) image of human glioblastoma tumor biopsy showing cells with numerous mitochondria with total-subtotal cristolysis and dysmorphic cristae (indicated by circles and ellipses). The presence of lipid droplets (indicated by white asterisks) is apparent and abundant. N indicates the nucleus. Magnification is at 4000 × and insert micrographs at 8000x. (Adapted from J Electron (Tokyo). 2008; 57:33–39)

Warburg based his hypothesis that ATP production through respiration was insufficient in cancer cells primarily on quantitative comparisons of oxygen consumption and lactate production between normal and cancerous tissues. Warburg’s evidence for OxPhos impairment in cancer was largely discounted based on Weinhouse’s 1976 statement: “*Despite massive efforts during the half-century following the Warburg proposal to find some alteration of function or structure of mitochondria, that might conceivably give some measure of support to the Warburg hypothesis, no substantial evidence has been found that would indicate a respiratory defect, either in the machinery of electron transport, or in the coupling of respiration with ATP formation, or in the unique presence or absence of mitochondrial enzymes or cofactors involved in electron transport”* (Weinhouse 1976). Based on the foundational principles of evolutionary biology and in recognition that mitochondrial structure determines function (Darwin 1859; Lehninger 1964; Bhargava and Schnellmann 2017; J. R. Friedman 2022; Brand et al. 1991; Brand and Nicholls 2011; Jezek et al. 2023; Miyazono et al. 2018; Seyfried 2012f; Pedersen 1978), the information in Fig. 2 and Table 1 presents substantial evidence for abnormalities in the number, structure, and function of mitochondria in all major cancers (Seyfried et al. 2020). Moreover, no tumor has yet been described with a normal content or composition of cardiolipin, the inner mitochondrial membrane-enriched phospholipid essential for the efficiency of OxPhos function (Kiebish et al. 2008; Venkatraman et al. 2024; J. Zhang et al. 2016). Reductions have also been reported in neoplasms for mitochondrial coenzyme Q, which like cardiolipin, is also essential for OxPhos efficiency (Sugimura et al. 1962; Shichiri et al. 1968; Battino et al. 1990; Alcazar-Fabra et al. 2016). Hence, these findings considered collectively address the premature criticisms of Weinhouse in providing a significant measure of support for Warburg’s central hypothesis.

**Table 1:** Mitochondrial abnormalities observed in common cancer

**Bladder cancer**: (Moriyama et al. 1984; Massari et al. 2016; Papadimitriou and Drachenberg 1994)
**Breast/Mammary cancers**: (Roskelley et al. 1943; Morciano et al. 2018; Ma et al. 2010; Santidrian et al. 2013; Pagano et al. 2014; Guha et al. 2018; Owens et al. 2011; Jogalekar and Serrano 2018; Elliott et al. 2012; Roddy and Silverberg 1980; Putignani et al. 2012, 2008; Rouiller 1960; Gadaleanu and Craciun 1987)
**Colorectal cancers**: (Roskelley et al. 1943; A. S. Sun et al. 1981; Modica-Napolitano et al. 1989; Piscitelli et al. 2003)
**Gliomas**: (Oudard et al. 1997; Feichtinger et al. 2014; G. J. Arismendi-Morillo and Castellano-Ramirez 2008; Katsetos et al. 2013; Deighton et al. 2014a; Deighton et al. 2014b; Sipe et al. 1973; Scheithauer and Bruner 1987a; Chinopoulos and Seyfried 2018; Mukherjee et al. 2019)
**Kidney/Renal cancer**: (Roskelley et al. 1943; Moreno et al. 2005; Sarto et al. 1997; Simonnet et al. 2003; Yusenko et al. 2010; Hervouet and Godinot 2006)
**Leukemias/lymphomas including AML, CLL and ALL**: (Roskelley et al. 1943; Morciano et al. 2018; Huhn 1984; Huhn et al. 1984; Kluza et al. 2011; Schumacher et al. 1975, 1974)
**Liver/Hepatic cancer**: (Cheuk and Chan 2001; Lo et al. 1968; Volman 1978; Rouiller 1960; Pedersen 1978; White et al. 1974; Cuezva et al. 2009, 2002; Capuano et al. 1996)
**Lung cancer**: (Morciano et al. 2018; Fernandez et al. 1976; Nicolescu and Eskenasy 1984a; Nicolescu and Eskenasy 1984b; Momcilovic et al. 2019)
**Melanoma**: (White et al. 1974; Hall et al. 2013; Taddei et al. 2012)
**Neuroblastoma**: (Brawn and Mackay 1980; Feichtinger et al. 2010; Morscher et al. 2015)
**Osteosarcoma**: (Hou-Jensen et al. 1972; van Waveren et al. 2006; B. Friedman and Gold 1968; Ghadially and Mehta 1970)
**Ovarian cancer**: (Andrews and Albright 1992; Ishioka et al. 2004; Dai et al. 2010)
**Pancreatic cancer**: (Huntrakoon 1983; Legrand and Pariente 1976; Novotny et al. 2013)
**Prostate cancer**: (Roskelley et al. 1943; Vayalil and Landar 2015; Mao et al. 1966)
**Retinoblastoma**: (C. N. Sun 1976; Nunes et al. 2015)
**Rhabdomyosarcomas**: (Bundtzen and Norback 1982; A. Li et al. 2018)
**Salivary Gland/Oral cancers**: (Kataoka et al. 1991; Kummoona et al. 2008)
**Uterine/Endometrial cancers**: (Ng et al. 1973; Cheuk and Chan 2001; Tang et al. 1979)

Evidence for abnormalities in mitochondrial number, structure or function in cancer as described previously in Seyfried et al. 2020

### Questionable Assumption 5: Fatty acid oxidation can provide sufficient ATP production through OxPhos in cancer cells

**Fig. 3:**
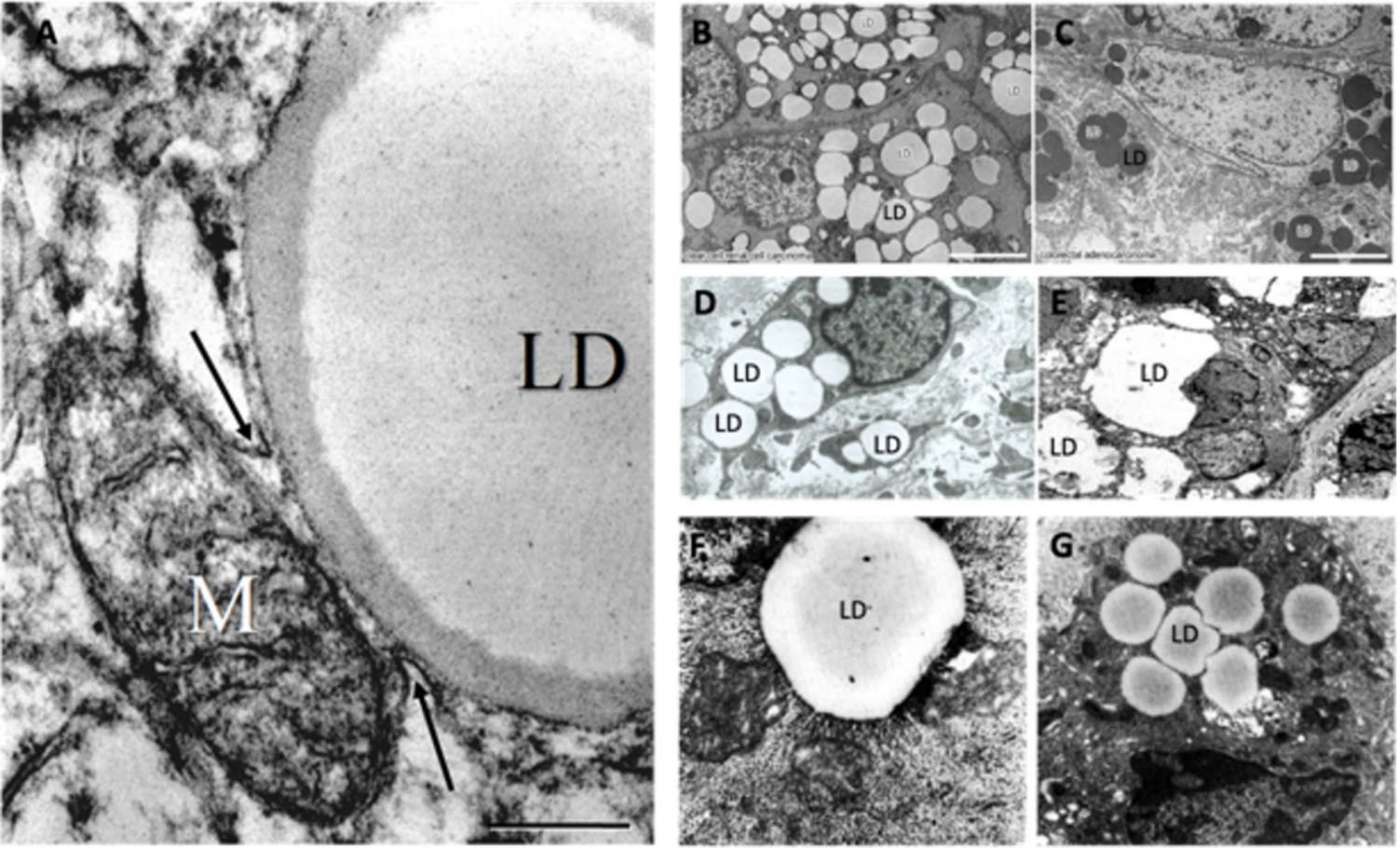
Lipid droplet accumulation in various malignant cancers. **A,** A large lipid droplet seen near abnormal mitochondria in glioblastoma (GBM). Mitochondria with cristae disarrangement and cristolysis. The interaction between lipid droplets and mitochondria is described as lipid droplet-associated mitochondria. Contact site (arrows). LD: Lipid droplet. M: mitochondria. Staining was uranyl acetate/lead citrate. Bar = 2.34 µm. Reprinted with permission from (G. Arismendi-Morillo 2011)**. B & C**. Electron microscopy showing cytoplasmic lipid droplets in renal clear cell carcinoma (**B**) and in colorectal adenocarcinoma (**C**). The lipid droplets are more electron dense in the colorectal adenocarcinoma sample than the renal cell carcinoma sample due to different processing. Bars = 5.0 µm. Images are from (Straub et al. 2010), and reprinted with permission through Creative Commons. **D & E**. Cytoplasmic lipid droplets in breast carcinoma. Electron micrograph of globular lipid droplets at 8000 × for C and D. Image is from (Guan et al. 2011) and reprinted with permission through Creative Commons. **F & G.** Ultrastructural observations of the lipid droplets in hepatoma showing close association of moderately osmiophilic lipid globules with mitochondria displaying irregular cristae and often containing electron-dense inclusions (38,000x). Reprinted through Creative Commons from (Freitas et al. 1990)

Despite substantial evidence showing that fatty acids are not a major fuel for driving the growth of malignant tumor cells (Bloch-Frankenthal et al. 1965; Holm et al. 1995; Ciaranfi 1938; Kuok et al. 2019; Lin et al. 2017; Ta and Seyfried 2015), the presence of cytoplasmic lipid droplets in various cancers has been considered evidence to many investigators that cancer cells can use fatty acid beta-oxidation for energy production and growth (Seyfried et al. 2024). It is well known that hypoxia-induced inhibition of OxPhos efficiency elicits the rapid formation of cytoplasmic lipid droplets in normal cells by blocking fatty acid beta-oxidation, (Niu et al. 2017; Seyfried et al. 2024; Gordon et al. 1977; Bhargava and Schnellmann 2017; Ralhan et al. 2021; S. J. Lee et al. 2013). Cytoplasmic lipid droplets also accumulate following induced abnormalities in mitochondria structure and function (Guerrieri et al. 2002; S. J. Lee et al. 2013; J. Liu et al. 2022a; Seyfried et al. 2024). If abnormalities in mitochondria structure and function have been documented in all major cancers, then cytoplasmic lipid droplets should also be observed in these same cancers. Indeed, cytoplasmic lipid droplets are seen in the most common cancer types where abnormalities in mitochondrial number, structure, and function are also seen (Tables 1 & 2). Electron microscopy images of lipid droplets from several different cancer types are presented in Figs. 2 & 3. The arrangement of the cancers in Table 1 with mitochondrial abnormalities is made to align with the arrangement of these same cancers with lipid droplet accumulation in Table 2 (Seyfried et al. 2020, 2024). The structural and functional abnormalities seen in cancer cell mitochondria would compromise OxPhos efficiency and thus contribute to the accumulation of triglyceride lipid droplets seen in cancer cell cytoplasm. Hence, the presence of cytoplasmic lipid droplets and the aerobic fermentation commonly seen in most malignant cancers can serve together as biomarkers for OxPhos inefficiency.

**Table 2:** Lipid droplets accumulation observed in common cancers

**Bladder cancers**: (Moriyama et al. 1984; Papadimitriou and Drachenberg 1994; Tirinato et al. 2021)
**Breast/Mammary cancers**: (Tirinato et al. 2021; Rouiller 1960; Zembroski et al. 2021; Borrego et al. 2021; Giudetti et al. 2019; Hershey et al. 2019; Jarc et al. 2018; Koizume and Miyagi 2016; Z. Li et al. 2020; Ramos and Taylor 1974; Guan et al. 2011)
**Colorectal/Gastric cancers**: (Accioly et al. 2008; Imazeki et al. 2021; Steuwe et al. 2014; Tirinato et al. 2021; M. Liu et al. 2022b; Koizume and Miyagi 2016; Z. Li et al. 2020; Straub et al. 2010)
**Gliomas**: (Bhatia et al. 2011; Taib et al. 2019; Geng et al. 2016; Hirose et al. 2001; Hoang-Minh et al. 2018; G. J. Arismendi-Morillo and Castellano-Ramirez 2008; Sipe et al. 1973; Scheithauer and Bruner 1987b; Korbecki et al. 2023; Offer et al. 2019; Kou et al. 2022; Maraqah et al. 2023; G. Arismendi-Morillo 2011)
**Kidney/Renal cancers**: (Thoenes et al. 1986; Dutta et al. 2016; Cruz et al. 2020; Wettersten et al. 2015; Petan et al. 2018; Koizume and Miyagi 2016; Lloreta-Trull and Serrano 1998; Z. Li et al. 2020; Straub et al. 2010; Hervouet and Godinot 2006)
**Leukemias/lymphomas including AML, CLL, and ALL**: (Bosc et al. 2020; Q. Chen et al. 2014; Hulse et al. 2021; Liang et al. 2023; J. Liu et al. 2022a; Thurgood et al. 2022; Tucci et al. 2021; Wu et al. 2022; Peiper et al. 1980; Yano et al. 2022)
**Liver/Hepatic cancer**: (Leduc and Wilson 1959; Lipsky et al. 1981; Petan et al. 2018; Noro et al. 2010; Z. Li et al. 2020)
**Lung cancer**: (Guo et al. 2013; C. Jin and Yuan 2020; Petan et al. 2018; Tirinato et al. 2017)
**Melanoma**: (Fujimoto et al. 2017; Giampietri et al. 2017; Nordenberg et al. 1987; Puskas et al. 2010)
**Neuroblastoma**: (Sainero-Alcolado et al. 2022; Zirath et al. 2013)
**Osteosarcoma**: (Ghadially and Mehta 1970; Roy et al. 2019; Garbe et al. 1981; Reddick et al. 1980)
**Ovarian cancer**: (Koizume and Miyagi 2016; Petan et al. 2018; Nieman et al. 2011; Z. Li et al. 2020; Iwahashi et al. 2021)
**Pancreatic cancer**: (Legrand and Pariente 1976; Sunami et al. 2017)
**Prostate cancer**: (Cruz et al. 2020; Z. Li et al. 2020; M. Chen et al. 2018; Mao et al. 1966)
**Retinoblastoma**: (Singh et al. 2016)
**Rhabdomyosarcomas**: (Sergi et al. 2012; Eyden 2010)
**Salivary Gland/Oral cancers**: (Brown and Aparicio 1984; He et al. 2022)
**Uterine/Endometrial cancers**: (Iwahashi et al. 2021; Sayers et al. 2021)

Evidence of lipid droplets in major cancers as previously described in Seyfried et al. 2024

Lipids can also act as uncoupling agents that produce oxidative stress in cells with inefficient or compromised OxPhos (Lehninger 1964; Zorov et al. 2014; Chance et al. 1979; Begriche et al. 2013; Massart et al. 2013; Ta and Seyfried 2015; Schonfeld and Reiser 2013). Lipid-induced uncoupling, however, might increase tumor growth by enhancing the use of fermentable fuels (glucose and glutamine) making it appear as if fatty acid beta-oxidation can provide sufficient ATP production through OxPhos for cancer cell growth (Zorov et al. 2014; Vozza et al. 2014; Giudetti et al. 2019; Seyfried et al. 2020). While some ATP production could be derived from fatty acid beta-oxidation in cancer cell mitochondria, it would be insufficient by itself to support the bioenergetic requirements of cancer cells. It is also plausible that some fatty acid beta oxidation could be anaplerotic for alpha-ketoglutarate (through oxaloacetate) when glutamate exits the mitochondria for transamination reactions. Furthermore, a risk of cell death from excessive production of fatty acid-derived reactive oxygen species (ROS) could be an outcome (Zorov et al. 2014). Hence, the data suggest that cancer cells store lipids in cytoplasmic droplets not as a fuel source for beta-oxidation, or for ATP production and growth, but rather as a protective mechanism to prevent oxidative stress and cell death and also to maintain cytoplasmic transaminations (Begriche et al. 2013; Massart et al. 2013; Seyfried et al. 2024; Ta and Seyfried 2015; Schonfeld and Reiser 2013).

### Questionable Assumption 6. Elevated substrate level phosphorylation (SLP) is required for the rapid growth of non-neoplastic cells

While glucose and glutamine-driven SLP in the cytosol and in the mitochondria, respectively, are necessary and sufficient for driving dysregulated cancer cell growth in vivo and in vitro, lipid-driven OxPhos appears to be the predominant driver of regenerating liver cells and colon crypt cells in vivo (Choi and Hall 1974; Caruana et al. 1986; Holecek 1999; Hague et al. 1997). It is interesting, however, that lipid droplets and hepatocyte swelling appear prior to OxPhos-driven liver cell regeneration suggestive of a transient dependency on SLP for ATP production (Guerrieri et al. 2002; Hu et al. 2023). In contrast to the glucose-driven growth of hepatoma cells, glucose inhibits the growth of regenerating liver hepatocytes (Capuano et al. 1997; Caruana et al. 1986; Burk et al. 1967). Although glucose and glutamine are also needed for regenerating liver cells, the levels are much less than those needed for proliferating cancer cells or normal cells grown in vitro (Z. Li et al. 2024). Unlike cancer cells where abnormalities in mitochondria structure and function are linked to a dependency on SLP for growth, the Crabtree effect can suppress OxPhos making the metabolism of non-neoplastic cells appear like that of tumor cells when grown in vitro (Clerici and Ciccarone 1965; Hague et al. 1997). Hence, caution is needed in recognizing the Crabtree effect as an in vitro artifact that enhances cytoplasmic SLP, while suppressing OxPhos efficiency, thus making energy metabolism in some cultured non-neoplastic cells appear like that seen in neoplastic tumor cells.

### Questionable Assumption 7: Somatic and germline mutations are responsible for the origin of cancer

Cancer (dysregulated cell growth) is widely considered a genetic disease based on findings of genomic abnormalities and vast numbers of mutations in oncogenes and tumor suppressor genes (Curtis 1965; Vogelstein et al. 2013; Stratton 2011; Hanahan and Weinberg 2011; Martinez-Jimenez et al. 2020; Nowell 2002; Gerstung et al. 2020). The National Cancer Institute has defined cancer as over 100 genetically distinct diseases thus solidifying the silent assumption that cancer is a genetic disease (http://www.cancer.gov/cancertopics/what-is-cancer) (Bell 1998). However, the absence of nuclear DNA mutations in some cancer cells (Brucher and Jamall 2016; Versteeg 2014; Sonnenschein and Soto 2000; Baker 2015; Greenman et al. 2007; Seyfried and Chinopoulos 2021; Rodrigues et al. 2016), the presence of cancer driver gene mutations in non-neoplastic normal tissues (Yizhak et al. 2019; Yokoyama et al. 2019; Pandya et al. 2024; Chanock 2018; Martincorena et al. 2018; Martinez-Jimenez et al. 2020; Ciwinska et al. 2024), together with data from the nuclear/mitochondrial transfer experiments (Israel and Schaeffer 1987; 1988; M. J. Kim et al. 2018; Fu et al. 2019; F. C. Kuo et al. 2024; C. Sun et al. 2019; Elliott et al. 2012; Seyfried 2015; Seyfried and Chinopoulos 2021; Kaipparettu et al. 2013; Yu et al. 2021), collectively represent irreconcilable inconsistencies challenging the somatic mutation theory as a credible explanation for the origin of cancer (Soto and Sonnenschein 2004, 2011; Baker 2015; Seyfried and Chinopoulos 2021; Hanselmann and Welter 2016). Even a single observation incompatible with a theory should question the validity of the theory; see pages 73–77 in (Kuhn 1957).

Land, Parada, and Weinberg showed that transforming primary embryonic fibroblasts required the cooperation between the *MYC* and the *RAS* oncogenes (Land et al. 1983b). Immortalization and transformation of non-malignant cells by mutant oncogene transduction has become a commonplace technique suggesting a direct experimental link between oncogene expression and tumorigenicity (Land et al. 1983a). However, the nuclear-cytoplasm transfer experiments demonstrated that oncogenic transformation is ultimately mediated by mitochondrial function, not by genetic drivers (Seyfried 2015; Seyfried and Chinopoulos 2021). Cytoplasmic (mitochondrial) factors were able to initiate tumorigenesis in the absence of nuclear drivers, and nuclear drivers did not initiate tumorigenesis in the presence of normal cytoplasmic factors (Seyfried 2015; Israel and Schaeffer 1987; 1988; Seyfried and Chinopoulos 2021). Moreover, the activation of the *K-ras G12V* mutation causes OxPhos insufficiency, increased ROS production, and increased cytosolic substrate level phosphorylation; findings that are more in line with the mitochondrial metabolic theory than with the somatic mutation theory (Hu et al. 2012; Seyfried and Chinopoulos 2021). Although specific somatic mutations may be considered secondary risk factors, they are neither necessary nor sufficient for tumorigenesis, while mitochondrial insufficiency coupled to compensatory upregulation of cytosolic and mitochondrial SLP appear both necessary and sufficient for tumorigenesis, independent of mutational background (D. C. Lee et al. 2024).

Additionally, no pathogenic germline cancer mutations have been found that are 100% penetrant (incomplete penetrance), meaning that most can be considered secondary risk factors rather than direct primary causes of cancer (Burgess et al. 2018; Marian 2014). Often, however, a gene with incomplete penetrance is not the primary cause of the disease, and likely, cancer-associated mutations (reported by the thousands) would follow this safe assumption. Among the highest penetrant are germline mutations in the *Tp53 g*ene (lifetime penetrance about 90%) found in people with the Li-Fraumeni syndrome (Malkin 2011). Penetrance is generally less for other germline cancer mutations including those for breast cancer (Shiovitz and Korde 2015; Risch et al. 2001), retinoblastoma (Otterson et al. 1997), Lynch syndrome (Wang et al. 2020), and most others (Qing et al. 2020). It is unclear how the somatic mutation theory could persist as a credible explanation for the origin of cancer considering the numerous inconsistencies with the theory (Sonnenschein and Soto 2000; Soto and Sonnenschein 2004; Seyfried and Chinopoulos 2021). It is also interesting that elevated ROS production and abnormalities in mitochondrial structure and function have been linked to several of the known cancer germline mutations e.g., the Li-Fraumeni syndrome (Y. Y. Kim et al. 2021; Matoba et al. 2006), the BRCA1 breast cancer (Q. Chen et al. 2020; Privat et al. 2014), retinoblastoma (Nicolay et al. 2015), and the Lynch syndrome (Rashid et al. 2019). Sequencing studies also show that cells with alterations in key driver genes, such as *Tp53*, are abundant in tissues of healthy individuals further complicating the association of driver gene mutations to neoplasia (Ciwinska et al. 2024). Similar to germline mutations that alter mitochondrial function, mutations in mitochondrial DNA (mtDNA) have also been found in some cancers leading Wallace and co-workers to suggest that cancer can best be defined as a type of mitochondrial disease (Petros et al. 2005). It should be recognized, however, that only certain mtDNA mutations are pathological or linked to neoplasia (Kiebish and Seyfried 2005; Cruz-Bermudez et al. 2015; Schon et al. 2012). As all major cancers express abnormalities in mitochondria structure and function, regardless of the presence or absence of gene mutations, we consider OxPhos inefficiency with compensatory SLP as the common metabolic phenotype of all major cancers.

### Cancer as a mitochondrial metabolic disease

We, like Warburg, consider the origin of energy (ATP) production as the central issue in cancer. Without energy no cell can remain viable or synthesize metabolites regardless of gene mutations or their connected signaling cascades. Major consumers of cellular energy are the membrane pumps including the sodium–potassium, the calcium, and the magnesium ATPases (Meyer et al. 2022; Veech et al. 2019; Hochachka and Somero 2002; Seyfried 2012c). Decreased ATP production in a cell by any means will cause a loss of K + , a gain of Na + and Ca + and, if persistent, to decreased voltage, altered volume, and cell death (Veech 1986). The energy of ATP hydrolysis is similar whether produced by cytosolic SLP in red blood cells, which lack mitochondria, or by OxPhos in mitochondrial containing tissues and is maintained in a very narrow band between − 56 and − 59 kJ/mol. Veech described the ΔG’ of ATP hydrolysis as the “still point in the turning world” (Veech et al. 2019). Although redox states for NAD(P) can vary appreciably, the ΔG’ of ATP remains within these narrow limits and underlies both genetic and metabolic processes (Veech et al. 2019). These processes are embodied in the second law of thermodynamics (Schneider and Sagan 2005).

According to traditional biochemistry, there are two primary mechanisms for producing cellular ATP. These include OxPhos and substrate level phosphorylation (SLP). OxPhos produces the majority of cellular ATP in normal cells through the F1-F0 ATPase which is linked to the mitochondrial electrochemical gradient. The amount of ATP produced through OxPhos is also linked to the structure and the protein/lipid composition of the cristae and the inner and outer mitochondrial membranes (Lehninger 1964; Zick et al. 2009; Cogliati et al. 2016; Colina-Tenorio et al. 2020; Glancy et al. 2020; Wallace 2005; Kiebish et al. 2008; G. Arismendi-Morillo et al. 2017; G. Arismendi-Morillo et al. 2012). SLP occurs at the kinase reactions in the pay-off phase of glycolysis in the cytosol, and through the succinyl-CoA synthetase reaction in the TCA cycle in the mitochondrial matrix. The succinyl-CoA synthetase reaction involves the transfer of a phosphate group from an amino acid of the synthetase itself to ADP (or GDP) to form ATP (or GTP) (Lancaster and Graham 2023; Majumdar et al. 1991; Lambeth et al. 2004). Pyruvate and succinate, together with ATP, are the products of the cytosolic pyruvate kinase M1 and the mitochondrial succinyl-CoA synthetase reactions, respectively. Under normal physiological conditions, the pyruvate is metabolized to acetyl CoA (and/or oxaloacetate) while the succinate is metabolized to fumarate. Both metabolites are fully oxidized to CO_2_ and water through metabolic reactions occurring within the TCA cycle of the mitochondrial matrix. Under hypoxic conditions, however, most of the pyruvate is metabolized to lactate while some of the succinate leaves the TCA cycle as both metabolites are produced as waste products of glucose-driven glycolysis and the glutamine-driven glutaminase pathways, respectively. Hence, the mechanism of ATP production in the presence of oxygen is the predominant difference between cancerous cells and non-cancerous cells.

**Fig. 4:**
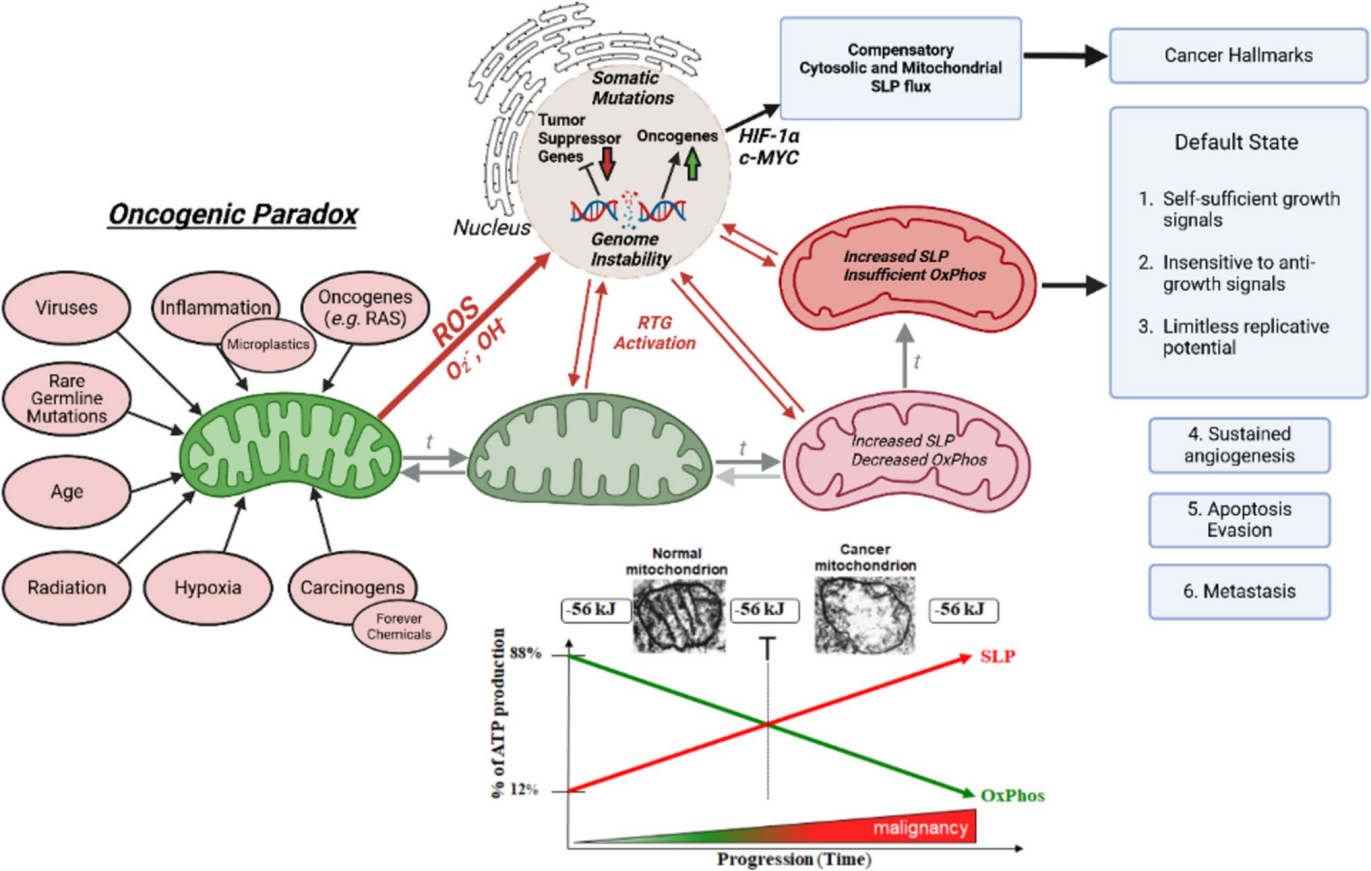
The origin of cancer as a mitochondrial metabolic disease. Cancer can arise from any number of unspecific risk factors in line with Szent-Gyorgyi’s “Oncogenic Paradox” (Szent-Gyorgyi 1977). Any one or combination of these oncogenic risk factors could cause OxPhos inefficiency thus increasing the production of reactive oxygen species (ROS), which would ultimately link to recognized hallmarks of cancer (Seyfried et al. 2014; Hanahan and Weinberg 2011; Seyfried and Shelton 2010; Seyfried and Chinopoulos 2021). The process by which each of these unspecific risk factors, which can also include recent findings on microplastics and forever chemicals (perfluorooctane sulfonate, per- and polyfluoroalkyl substances, etc.) can cause chronic OxPhos insufficiency (Seyfried 2012b; d; e; g; Seyfried and Chinopoulos 2021; S. Li et al. 2023; Y. Liu et al. 2023; Hofmann et al. 2023). Excessive production of ROS (OH^−^ and O_2_^.−^) is carcinogenic and mutagenic and would cause significant damage to lipids, proteins, and nucleic acids in both the mitochondria and in the nucleus (Zhu et al. 2018). Nuclear genomic instability, including the vast array of somatic mutations and aneuploidy, would arise as a consequence of ROS damage together with chronic extracellular acidification and inflammation through a bidirectional interaction between the provocative agent and cells within a tissue (Sonugur and Akbulut 2019; Seyfried 2012a; Seyfried et al. 2014, 2017; Seoane et al. 2011). Indeed, mutations in the *p53* tumor suppressor gene and genomic instability have been linked directly to OxPhos insufficiency and mitochondrial ROS production in cancer stem cells (Matoba et al. 2006; Bartesaghi et al. 2015). Fermentation metabolism and ROS formation underlie the hyperproliferation of tumor cells as efficient OxPhos is necessary for maintaining the differentiated state of cells (see text for details). A gradual reduction in OxPhos efficiency would elicit a mitochondrial stress response through retrograde (RTG) signaling (Seyfried 2012g; Srinivasan et al. 2016; Ryan and Hoogenraad 2007; Biswas et al. 2008). RTG activation would cause persistent expression of various oncogenes, e.g., *Hif-1a* and c-*Myc*, that upregulate receptors and enzymes in both the glycolysis and the glutaminolysis pathways necessary to compensate for OxPhos insufficiency and to maintain the viability of incipient cancer cells. (Wise et al. 2008; Dang et al. 2009; Dang and Semenza 1999; D. Yang and Kim 2019; Semenza 2017; Srinivasan et al. 2016). Oncogenes therefore become facilitators of increased cytosolic and mitochondrial substrate level phosphorylation (SLP) that drive dysregulated growth in cells with insufficient OxPhos. Glutamine-driven ATP production through mitochondrial SLP in the glutaminolysis pathway will compensate for lost ATP production through OxPhos or from PKM2 expression in the glycolytic pathway (Seyfried et al. 2020; Chinopoulos 2020; D. C. Lee et al. 2024). The path to carcinogenesis will occur only in those cells capable of sustaining energy production through SLP. Despite the shift from respiration to SLP, the ∆G′ATP hydrolysis remains constant at approximately −56 kJ indicating that the energy from SLP compensates for the reduced energy from OxPhos. Metastasis arises from respiratory damage in cells of myeloid/macrophage origin as described in the text. Tumor progression and degree of malignancy is linked directly to ultrastructure abnormalities (mitochondrial cristolysis) and to the energy transition from reduced OxPhos to increased cytosolic and mitochondrial SLP (Seyfried et al. 2020; D. C. Lee et al. 2024; Ravasz et al. 2024; Doczi et al. 2023; G. Arismendi-Morillo et al. 2017). The *t* represents the fission–fusion-mitophagy cycle that modulates the mitochondrial network and is disrupted in cancer (Boulton and Caino 2022; H. Yang et al. 2021). The **T** signifies an arbitrary threshold when the shift from OxPhos to SLP would become irreversible. The linkage of SLP to malignancy is as solid as that of gravity to the redshift (Seyfried et al. 2020). This scenario links major cancer hallmarks to an extrachromosomal and epigenetic respiratory dysfunction thus solving the oncogenic paradox. Reprinted with modifications from (Seyfried and Shelton 2010; Seyfried et al. 2020). Figure created using BioRender

**Fig. 5:**
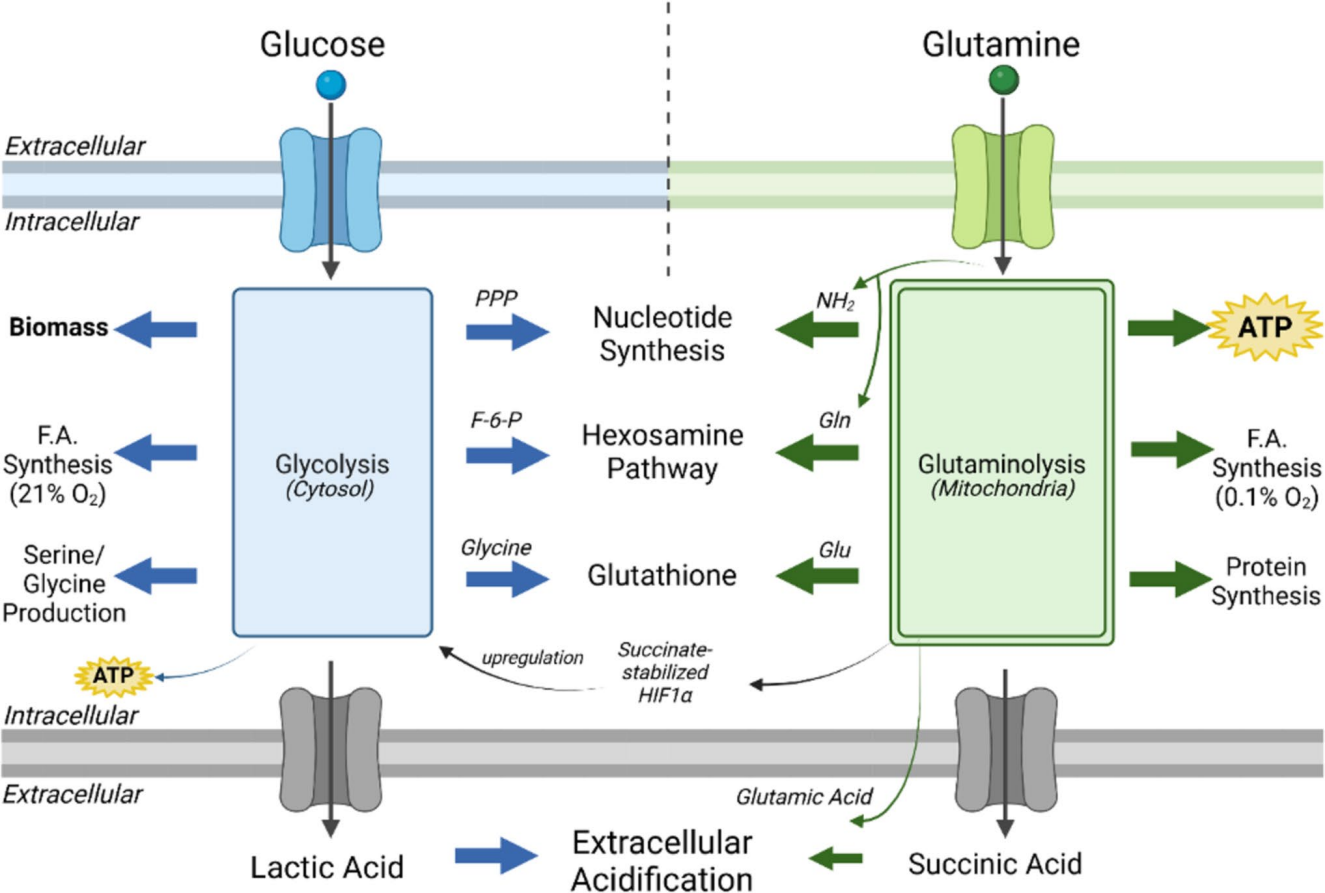
High-throughput synergy between the glycolysis and the glutaminolysis pathways drive the dysregulated growth of glioma cells. Glucose (blue) is metabolized through the 10-step glycolytic pathway and contributes to several pro-biomass pathways such as: nucleotide synthesis via the pentose phosphate pathway (PPP), diverting fructose-6-phosphate (F-6-P) toward the hexosamine pathway, and glycine to produce glutathione. Some glucose carbons are diverted to synthesize fatty acids in normoxia. Glucose carbons that reach pyruvate kinase are exported from the cell as lactate. Glutamine (green) enters the glutaminolysis pathway. Glutamine is essential for producing glucosamine-6-phosphate, a key intermediate in the hexosamine pathway that contributes to N- and O-linked glycosylation. The amide nitrogen released from the conversion of glutamine to glutamate contributes to nucleotide synthesis. Glutamate is combined with glycine and cysteine to form glutathione to act as an antioxidant. The remaining glutamate is converted first to alpha-ketoglutarate (a-KG). a-KG will divert in the reductive TCA cycle through citrate and be used for fatty acid synthesis in hypoxia (Ta and Seyfried 2015). Otherwise, a-KG follows the oxidative pathway and is converted to succinyl-CoA. Succinyl-CoA is the substrate for mitochondrial substate level phosphorylation (mSLP) that produces ATP and succinate (Chinopoulos and Seyfried 2018). Succinate has been shown to stabilize HIF1a via inhibition of prolyl hydroxylase (Selak et al. 2005), a key protein that upregulates glycolysis. The excretion of both succinate and glutamate into the extracellular matrix, together with lactate excretion, contribute to the acidification of the microenvironment. All major hallmarks of cancer can be linked to chronic OxPhos insufficiency coupled to the protracted upregulation of SLP (Seyfried and Chinopoulos 2021). Figure created using BioRender (D. C. Lee et al. 2024)

The linkage of SLP to cancer malignancy is shown in Fig. 4**,** while Fig. 5 illustrates the synergy between the glycolysis and the glutaminolysis pathways, which facilitate biomass synthesis and ATP production in brain tumor cells. Cancer is rare in cells that cannot chronically replace ATP production through OxPhos with ATP production through SLP, e.g., post-mitotic cardiac myocytes and brain neurons. While these cells can rapidly upregulate ATP production through SLP under acute oxygen deficiency, e.g., cardiac arrest or epileptic seizures, they cannot sustain this ATP production for more than a few minutes without suffering catastrophic death thus preventing a protracted transition to substrate level phosphorylation. In contrast to the transient accumulation of lactate and succinate under hypoxia in normal cells, the waste products of glucose and glutamine fermentation continue to accumulate in cancer cells even in the presence of oxygen. The persistent extracellular accumulation of lactate and succinate together with the cytoplasmic accumulation of lipid droplets in cancer cells result in large part from the well documented abnormalities in the number, structure, and function of mitochondria. It should also be recognized that the mitochondrial proton motive force controls calcium signaling, which regulates cyclins, the cell cycle, and the quiescent or differentiated state of the cell (Arciuch et al. 2012; Casanova et al. 2023; Horbay and Bilyy 2016; Kumar Sharma et al. 2022; Osellame et al. 2012; Zheng et al. 2023). In other words, it is the efficiency of OxPhos that maintains the differentiated state of somatic cells while the chronic loss of OxPhos efficiency leads to SLP-driven dysregulated cell growth, i.e. neoplasia. Just as proliferation is the default state of metazoan cells, SLP is the default energetic state of cells under reduced or absent oxygen (Szent-Gyorgyi 1977; Soto and Sonnenschein 2004). We also solved Szent-Gyorgyi’s “oncogenic paradox” in showing that chronic OxPhos insufficiency coupled to increased SLP is the common pathophysiological mechanism linking malignant transformation to a broad range of unspecific influences including age, intermittent hypoxia, carcinogens, localized and systemic inflammation, radiation, rare germline mutations, oncogenic viruses, etc. (Szent-Gyorgyi 1977, Seyfried 2012e, Seyfried, Flores et al. 2014, Seyfried and Chinopoulos 2021). Hence, a greater dependency on substrate level phosphorylation than on OxPhos for energy is the pathophysiological phenotype common to all major cancers.

### Therapeutic implications

According to the American Cancer Society almost 612,000 people are projected to die from cancer in the US in 2024, which amounts to about 1,700 people dying/day or about 70 people dying/hour (Siegel et al. 2024). The anti-smoking campaign of the 1990’s was largely responsible for preventing the number of yearly cancer deaths from being even higher (Siegel et al. 2024). The failure to reduce cancer deaths results in large part from the persistent belief that cancer is a genetic disease according to the somatic mutation theory (Soto and Sonnenschein 2004; Seyfried and Chinopoulos 2021). The “Press Pulse” therapeutic strategy for cancer management was developed based on the new understanding that cancer is a disorder of mitochondrial energy metabolism (Seyfried et al. 2017; Duraj et al. 2024). The strategy involves the simultaneous restriction of glucose and glutamine while the body is placed in a state of nutritional ketosis.

The ketone body, beta-hydroxybutyrate, has been designated a “super fuel” because it, a) does not uncouple the electrochemical gradient like fatty acids and thus increases the ΔG’ of ATP hydrolysis, b) has more carbon-hydrogen bonds than pyruvate, c) produces few oxygen radicals during its metabolism, and d) can replace glucose as an energy source for the brain and other organs, (Veech et al. 2001; Veech 2004; Cahill and Veech 2003). Indeed, Drenick and co-workers showed that glucose concentrations as low as 0.5 mmoles/liter (9 mg/100 ml) failed to precipitate hypoglycemic reactions in insulin-treated subjects when their circulating levels of beta-hydroxybutyrate was elevated (Drenick et al. 1972). The whole-body transition from glucose to ketone bodies will reduce availability of glucose to both the glycolytic and pentose phosphate pathways while also consuming some of the CoA needed for driving mSLP (Figs. 1 & 5). Hence, the reduction of circulating glucose and elevation of ketone bodies will deprive tumor cells of energy, and the glucose carbons needed for the synthesis of growth metabolites (Boros et al. 1998; Mazat 2021).

Press-pulse ketogenic metabolic therapy (KMT), involving the simultaneous targeting of cytosolic and mitochondrial SLP in tumor cells while enhancing OxPhos efficiency in nontumorigenic normal body cells, offers a therapeutic strategy for managing most cancers (Winter et al. 2017; Seyfried et al. 2017; Duraj et al. 2024). KMT will also reduce the lactate/succinate-acidification of the tumor microenvironment, which can block the efficacy of immunotherapies (Heuser et al. 2023). The glucose/ketone index (GKI) was developed for estimating the degree of therapeutic ketosis by measuring the mM ratio of glucose to ketones (beta-hydroxybutyrate) in the circulation (Meidenbauer et al. 2015; Duraj et al. 2024). Therapeutic ketosis will enhance OxPhos efficiency in normal cells while lowering glucose availability to tumor cells. In general, the lower is the GKI the slower is the tumor growth (Seyfried et al. 2003; Seyfried et al. 2021; Akgoc et al. 2022). This is important because aggressive tumor growth and poor patient survival are linked to elevated blood glucose levels in a variety of cancers (McGirt et al. 2008; Ramteke et al. 2019; P. Zhang et al. 2022; Santos and Hussain 2020). KMT can also be used together with other therapies, including standards of care, if efficacy can be maintained with no or minimal toxicity (Duraj et al. 2024; Kiryttopoulos et al. 2025).

While the elevation of circulating ketone bodies allows for the chronic restriction of glucose availability, glutamine availability cannot be chronically restricted due to its importance for the urea cycle, gut health, and immune system function (Seyfried et al. 2017; Duraj et al. 2024). Consequently, glutamine targeting should be pulsed rather than pressed to avoid adverse toxic effects. Cells of the immune system, especially macrophages, use glutamine for energy and other biological functions including wound healing (P. Newsholme 2001; Wculek et al. 2022; Hofer et al. 1999). Most metastatic cancers also express biomarkers of macrophages indicating a macrophage/myeloid origin of metastatic cancer arising from either a direct transformation of macrophage/myeloid cells or from fusion hybridizations between neoplastic stem cells and macrophages (Seyfried and Huysentruyt 2013; Huysentruyt et al. 2008; Huysentruyt and Seyfried 2010; Ruff and Pert 1984; Lindstrom et al. 2017; Lopez-Collazo and Hurtado-Navarro 2025; Powell et al. 2011; Pawelek 2014; Schramm 2014). In other words, the same fuel needed for driving metastasis is also needed for supporting immune cell function (Rodrigues et al. 2016; Shelton et al. 2010; Mukherjee et al. 2019; Duraj et al. 2024; Seyfried et al. 2017). These findings indicate that strategic glucose & glutamine targeting will be necessary for managing invasive and metastatic cancers while maintaining normal immune cell function.

It is also interesting that some parasites, like tumor cells, rely more heavily on SLP than on OxPhos for ATP production (Bochud-Allemann and Schneider 2002; Kita et al. 2002; Saz 1981). These findings could make parasite medications potentially non-toxic, cost-effective treatments for managing cancer including pediatric high-grade gliomas (Veerakumari and Munuswamy 2000; Xiao et al. 1994; Gallia et al. 2021; Hunger-Glaser et al. 1999; Mukherjee et al. 2023). While some have suggested that targeting OxPhos might also be effective in managing cancer, serious toxicity to normal cells could be an unanticipated consequence of such therapeutic strategies (Alcala et al. 2024; Greene et al. 2022; X. Zhang and Dang 2023). It is our view that the simultaneous targeting of glucose and glutamine while transitioning the body to nutritional ketosis will stress tumor cells of the energy and the carbons and nitrogen needed for the synthesis of growth metabolites. This therapeutic strategy will also reduce the acidification and inflammation in the tumor microenvironment thus facilitating the non-toxic metabolic management of cancer (Boros et al. 1998; Mazat 2021; Seyfried et al. 2017; Duraj et al. 2024).

## Conclusions

Scientific theories are simply attempts to explain the facts of nature. Reality is based on replicated facts, whereas the interpretation of the facts is based on credible theories (Seyfried and Chinopoulos 2021). While Warburg was largely correct in recognizing OxPhos insufficiency linked to compensatory lactic acid fermentation as the origin of cancer, several questionable assumptions and measurements of cellular ATP production have confounded data interpretation linked to his hypothesis. Warburg’s reliance on oxygen consumption rate and lactate production as measures to support his hypothesis were inaccurate and contributed to confusions in biochemical terminologies, which persist even today in the cancer metabolism field. Moreover, he did not know that glutamine-driven mitochondrial SLP through the glutaminolysis pathway could also contribute to cancer cell ATP production. These issues have now been better clarified. While the somatic mutation theory is currently the predominant explanation for the origin of cancer, the mitochondrial metabolic theory offers a more credible explanation that can lead to more effective and less toxic therapeutic strategies for managing cancer.

## Data Availability

No datasets were generated or analysed during the current study.

## Abbreviations

OxPhos: Oxidative phosphorylation
SLP: Substrate level phosphorylation
ROS: Reactive oxygen species
mSLP: Mitochondrial substrate level phosphorylation
SMT: Somatic mutation theory
MMT: Mitochondrial metabolic theory
KMT: Ketogenic metabolic therapy
TCA: Tricarboxylic acid
